# Supplementary material for: Silicon–Gold Nanoparticles Affect Wharton’s Jelly Phenotype and Secretome during Tri-Lineage Differentiation
Source: Int J Mol Sci. 2022 Feb 15;23(4):2134. doi: 10.3390/ijms23042134 (PMC8874983; doi:10.3390/ijms23042134)
Supplement: Supplementary file 1 [file ijms-23-02134-s001.zip › ijms-1561456-supplementary.pdf]

Table S1. Humoral factors measured in MSCs supernatants

	Growth factors	Chemokines	Cytokines
	**G-CSF**	**Fractalkine/CX3CL1**	**IL-4**
Produced by	**VEGF**	**GRO/CXCL1**	**IL-6**
Warthon's Jelly	**GM-CSF**	**IL-8/ CXCL8**	
MSCs	**PDGF-AA**	**IP10/ CXCL10**	
	**FGF-2**	**MCP-1/CCL2**	
		**MCP-3/ CCL7**	
		**RANTES/CCL5**	
	sCD40L	MDC/ CCL22	TNF-α; β
	EGF	MIP-1α/ CCL3	IFNα2; γ
Not produced	FLT-3L	MIP-1β/ CCL4	TGFα
	PDGF-AB/BB	Eotaxin/ CCL11	IL-1RA
			Interleukins: 1α;β, 2, 3, 5, 7, 9, 10, 12p40, 12p70, 13, 15, 17A

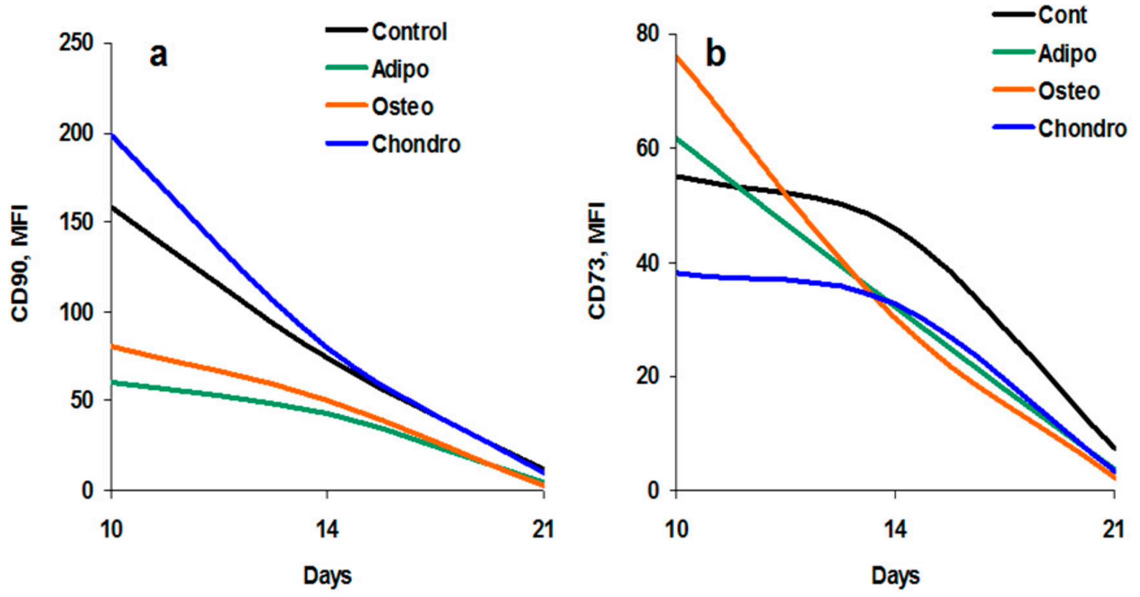

Figure S1. Time-dependent expression of MSCs surface markers during adipogenic (Adipo), Osteogenic (Osteo), and chondrogenic (Chondro) differentiation estimated by flow cytometry. MFI designate mean fluorescence intensity. Data are presented for CD90 (a) and CD73 (b) expression. Results are pooled from 2 experiments.

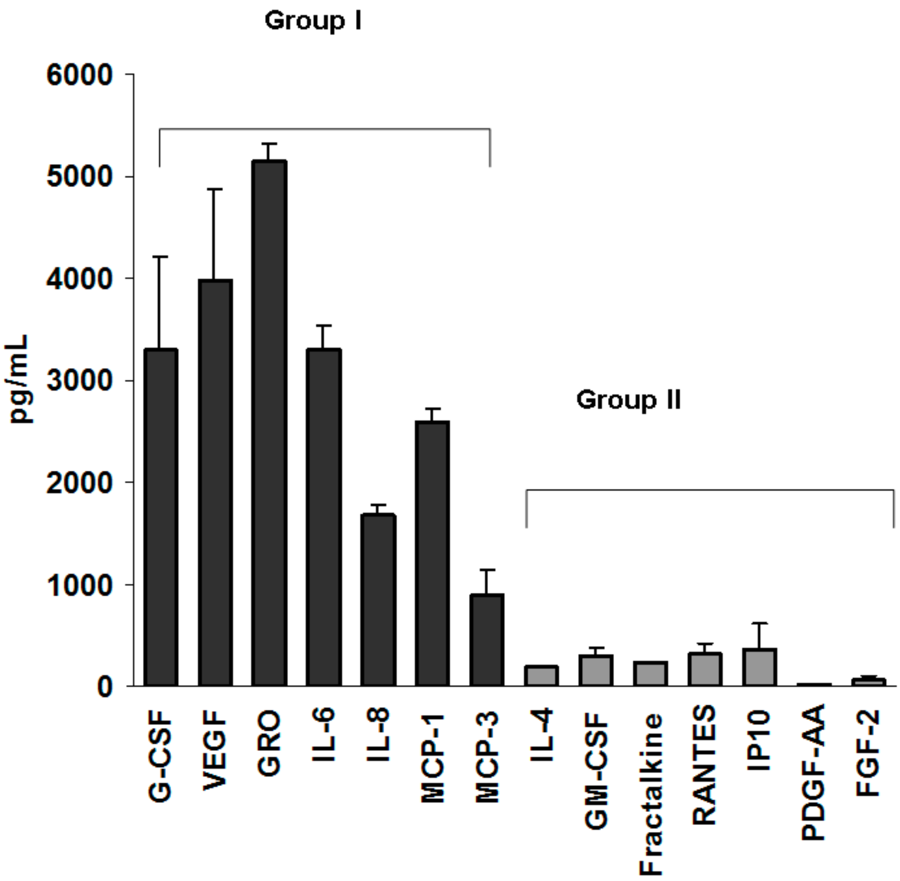

Figure S2. Concentration of humoral factors stably produced by MSCs. Results are pooled from 2 experiments.

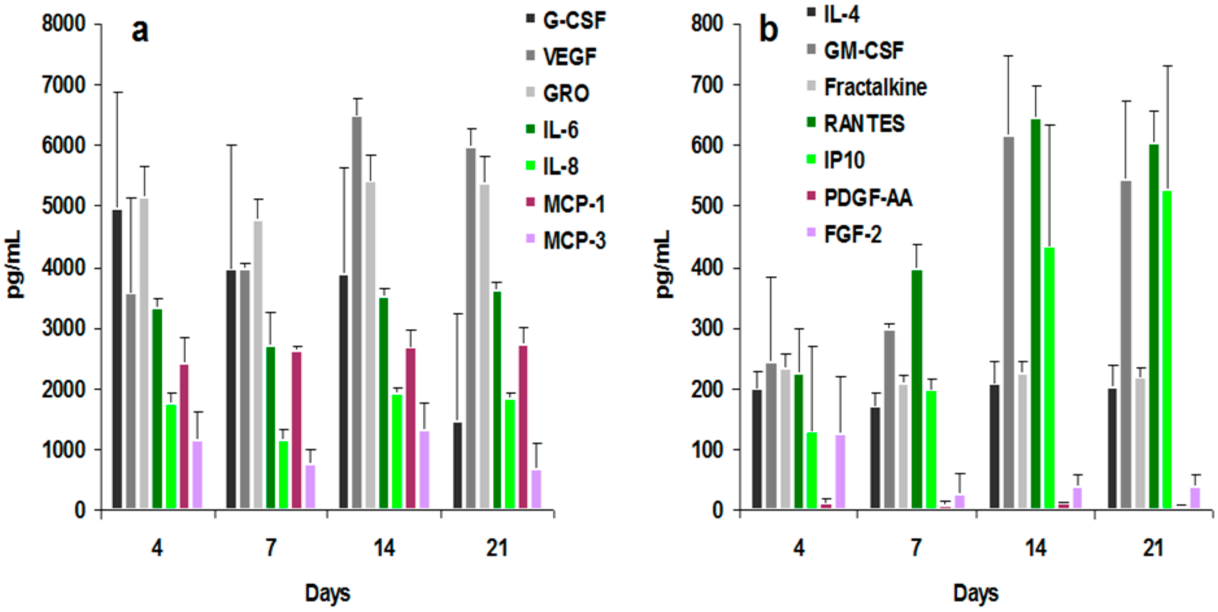

Figure S3. Time-dependent humoral factor production by unstimulated MSCs.

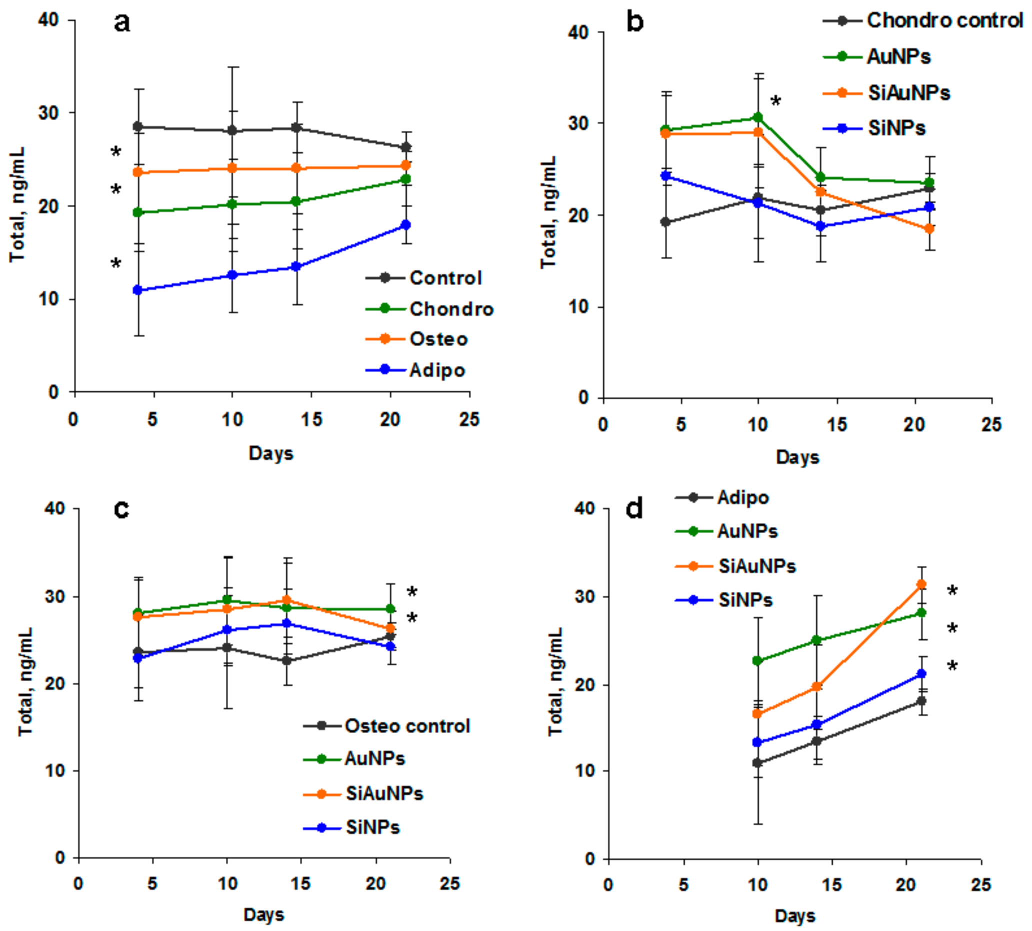

Figure S4. Total concentration of humoral factors during MSCs differentiation without NPs (a) and in the presence of NPs during condrogenic (b), osteogenic (c), and adipogenic (d) differentiation. Significant differences are shown with asterisks.

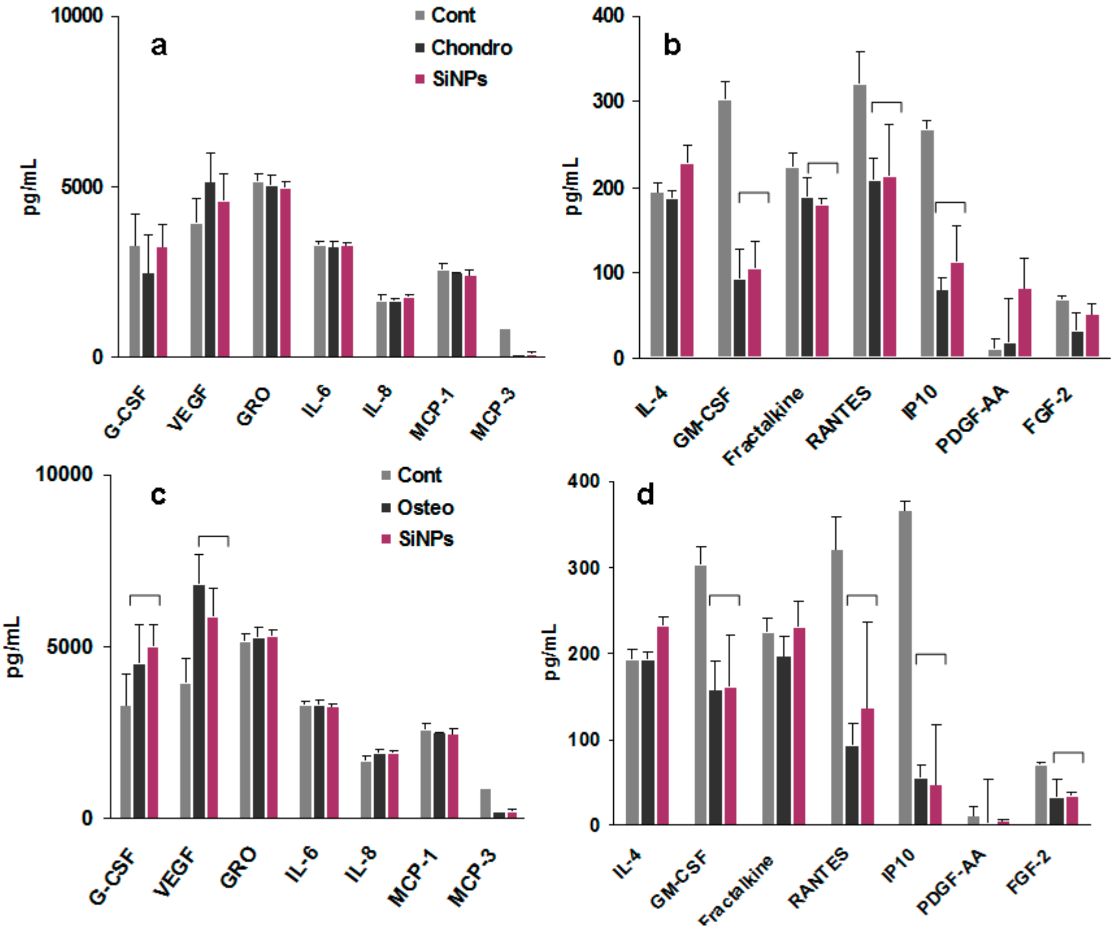

Figure S5. Effect of SiNPs on soluble factor production by MSCs at 14th day of chondrogenic (a) and osteogenic (b) differentiation. Common effects of differentiating stimuli and SiNPs are shown by brackets.
